# Small molecules inhibit growth, viability and ergosterol biosynthesis in *Candida albicans*

**DOI:** 10.1186/2193-1801-2-26

**Published:** 2013-01-29

**Authors:** Sandeep B Rajput, S Mohan Karuppayil

**Affiliations:** DST-FIST and UGC-SAP Sponsored School of Life Sciences, SRTM University, Nanded, 431-606, M.S India

**Keywords:** Small molecules, Candida albicans, Ergosterol biosynthesis, Antifungals

## Abstract

The aim of this work was to evaluate the anti-*Candida* efficacy of twenty five molecules of plant origin. Based on their MICs, effective molecules were categorized into four categories. Susceptibility testing of test compounds was carried out by standard methodology (M27-A2) as per CLSI guidelines. Minimum Fungicidal Concentration (MFC) was determined as the lowest concentration of drug killing 99.9% cells. Effect on sterol profile was evaluated by sterol quantitation method. Among the screened molecules, cinnamaldehyde, piperidine, citral, furfuraldehyde and indole were potent inhibitors of growth and viability. Exposure of *Candida* cells to cinnamaldehyde, piperidine, citral, furfuraldehyde, indole, α- and β- pinene at MIC’s, altered ergosterol profile. Our results indicate that the molecules altering sterol profile may exert their antifungal effect through inhibition of ergosterol biosynthesis and could be good candidates for fungal specific drug development.

## Background

*Candida albicans* is a predominant organism associated with candidiasis and can lead to severe diseases which range from superficial infections to life threatening systemic disorders (Kaleli et al. 2007). *Candida* species are now recognized as a major cause of hospital-acquired infections (Maschmeyer 2006; Berman & Sudbery 2002). Most of the drugs available to treat *Candida* infections target the ergosterol biosynthetic pathway or its end product ergosterol. The antifungal agents which target ergosterol include azoles, allylamines, thiocarbamates, polyenes and morpholines (Sanglard et al. 2003). Most of the antifungals have severe hepatotoxicity, nephrotoxicity and in addition human pathogenic fungi have also developed resistance (Cannon et al. 2009; Odds et al. 2003; Gupta & Thomas 2003). The high toxicity and multiple drug resistance associated with various standard antifungals have necessitated the search for safer alternatives.

In recent years, secondary metabolites have been extensively investigated as sources of medicinal agents (Michal & Klaus 2009). Molecules of natural origin (e.g. plants) are known to possess good antifungal potential (Cowan 1999) and may efficiently target various biosynthetic pathways like ergosterol synthesis. Essential oils as well as their components have been shown to exert anti-*Candida* activities (Zore et al. 2010,2011; Devkatte at al. 2005). Small molecules such as carvacrol, thymol, eugenol and epigallocatechin-3-gallate (EGCG) are reported to inhibit ergosterol biosynthesis in *C. albicans* (Ahmed et al. 2011; Ahmad et al. 2010; Navarro-Martinez et al. 2006).

In this communication we report the anti-*Candida* properties of twenty five small molecules of plant origin.

## Results

### Planktonic growth of C. albicans inhibited by natural molecules

Majority of the molecules were found to be effective and showed considerable inhibition of growth at very low concentration. On the basis of MIC achieved against *C. albicans* ATCC 90028, molecules could be classified into four classes. Molecules, showing MIC at 0.0625-0.5 mg/ml concentration are classified as most effective (ME). Molecules with an MIC ranging from 1 to 2 mg/ml are included in the moderately effective (MoE) group. Molecules with an MIC of 2 to 4 mg/ml are considered as less effective (LE). While, molecules which did not show MIC up to highest concentration tested (i.e. 8 mg/ml) are non effective (NE) (Table 1).

**Table 1:** Effect of various molecules on growth, viability and ergosterol biosynthesis in *C. albicans*

Group	Molecules	MIC (mg/ml)	MFC (mg/ml)	% decrease in ergosterol content of ***C. albicans*** at their respective MIC value
Most effective (0.0625-0.5 mg/ml)	cinnamaldehyde	0.0625	0.25	58 (± 0.502)
	piperidine	0.25	1	60 (± 0.123)
	indole	0.5	1	55 (± 0.652)
	furfuraldehyde	0.5	1	96 (± 0.926)
	citral	0.5	1	99 (± 0.865)
Moderately effective (1–2 mg/ml)	beta-Pinene	1	1	90 (± 0.325)
	salicylic acid	1	NA	NE
	guaiacol	2	2	NE
	cymene	2	8	NE
Less effective (4–8 mg/ml)	caffeine	4	NA	NE
	camphene	4	4	NE
	citronellol	4	8	NE
	geraniol	4	NA	NE
	geranyl acetate	4	NA	NE
	alpha-Pinene	8	NA	40
	carvone	8	NA	NE
	linalool	8	NA	NE
	thujone	8	NA	NE
Non effective	bisabolol	NA	NA	NE
	jasmonate	NA	NA	NE
	isopulegol	NA	NA	NE
	limonene	NA	NA	NE
	1,4-cineole	NA	NA	NE
	1,8-cineole	NA	NA	NE
	menthol	NA	NA	NE

**(NA:** MIC not achieved at highest conc. tested i.e. 8 mg/ml; **NE:** Not effective; values in parenthesis in last column represent the mean ±SD of three experiments**).**

**Figure 1:**
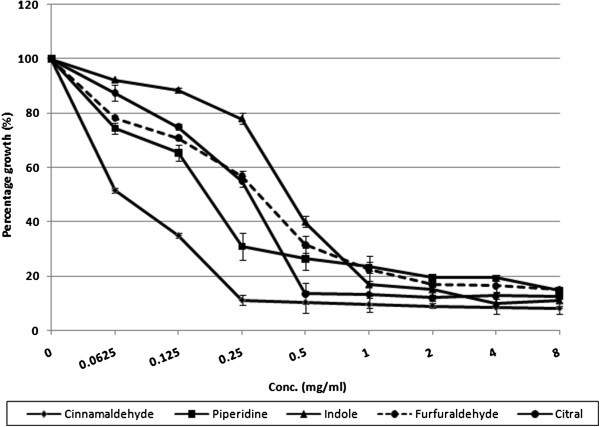
**Effect of ‘most effective’ molecules on growth of*C. albicans*ATCC 90028.**

**Figure 2:**
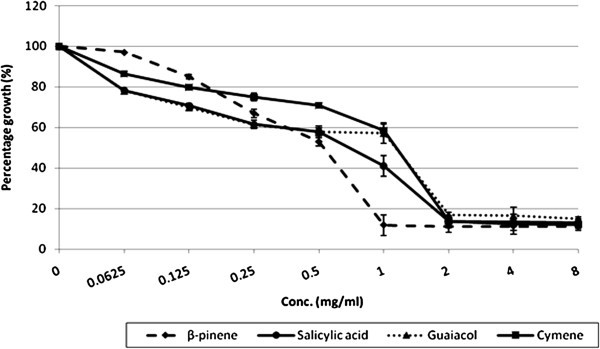
**Effect of ‘moderately effective’ molecules on growth of*C. albicans*ATCC 90028.**

**Figure 3:**
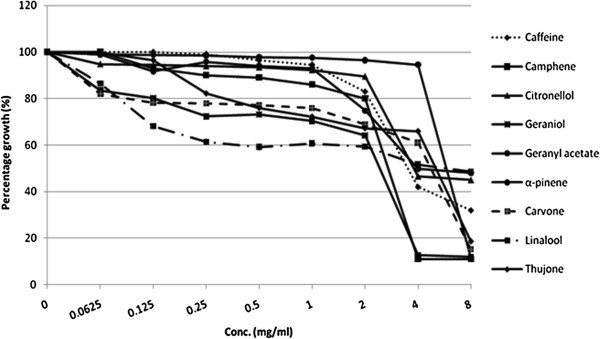
**Effect of ‘less effective’ molecules on growth of*C. albicans*ATCC 90028.**

Five molecules of the screened were most effective (Figure 1). Cinnamaldehyde was the best, with an MIC of 0.0625 mg/ml. Piperidine showed promising growth inhibitory activity at 0.25 mg/ml. Growth inhibition by citral, furfuraldehyde, and indole was noted at 0.5 mg/ml. β-pinene and salicylic acid were the best among the group of four moderately effective members, causing growth inhibition at 1 mg/ml. Guaiacol and cymene exhibited MIC at 2 mg/ml (Figure 2). Nine were found to be less effective. Among these molecules caffeine, camphene, citronellol, geraniol and geranyl acetate, inhibited growth at 4 mg/ml. The remaining four molecules namely α-pinene, carvone, linalool and thujone were not having significant effect on growth up to 4 mg/ml, whereas MIC was achieved at 8 mg/ml (Figure 3). Rest seven molecules, including jasmonate, bisabolol, isopulegol, limonene, 1,4 -cineole, 1,8 -cineole and menthol, failed to inhibit growth even at 8 mg/ml. Fluconazole showed MIC at 2 μg/ml. Since fluconazole is fungistatic MFC can’t be achieved.

### Killing effect of molecules on C. albicans

Effect of small molecules on viability of *C. albicans* was determined in terms of minimum fungicidal concentration (MFC). Among the molecules tested, cinnamaldehyde was the most efficient inhibitor of *C. albicans* viability at 0.25 mg/ml. Four of the ‘most effective’ group have shown excellent candida-cidal activity at 1 mg/ml which caused killing of all cells, including piperidine, indole, fufuraldehyde and citral. β-pinene from the ‘moderately effective’ group exhibited cidal effect at 1 mg/ml. Absolute killing of cells by guaiacol and camphene was achieved at 2 and 4 mg/ml respectively. Fungicidal activity of cymene and citronellol for *C. albicans* was 8 mg/ml. Whereas no MFC could be achieved for geraniol, geranyl acetate, salicylic acid, jasmonate,α-pinene, caffeine, bisabolol, isopulegol, limonene, 1,4-cineole, 1,4-cineole, menthol, carvone, linalool and thujone up to 8 mg/ml (Table 1).

### Exposure to molecules altered sterol profile in C. albicans

**Figure 4:**
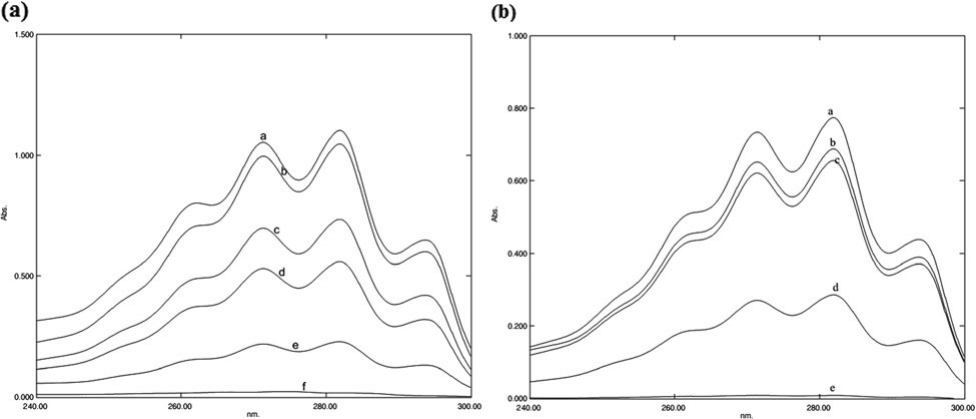
**UV spectrophotometric sterol profiles of*Candida albicans*were grown for 16 h in Sabouraud dextrose broth containing 0.5 (curve b), 0.25 (curve c), 0.125 (curve d), 0.0625 (curve e), 0.0313 (curve f) mg/ml of Furfuraldehyde (a); 0.5 (curve b), 0.25 (curve c), 0.125 (curve d), 0.0625 (curve e), mg/ml of Citral (b) and 0 mg/ml of drug (curve a), sterols were extracted from cells, and spectral profiles between 240 and 300 nm were determined.**

All molecules of the ‘most effective’ group significantly (*p* < 0.05) altered sterol profile of *C. albicans* in a concentration dependant manner. Among the molecules tested, citral and furfuraldehyde were most effective at their MIC’s and caused 99 to 96% reduction in total ergosterol content respectively (Figure 4). The percent decrease in total cellular ergosterol content was 58, 60 and 55% in presence of MIC’s of cinnamaldehyde, piperidine and indole respectively. β- pinene from the ‘moderately effective’ group, caused 90% reduction in ergosterol content at 1 mg/ml concentration. At 8 mg/ml concentration, α- pinene reduced 40% of total ergosterol content.

## Discussion

In the present study, we have examined the efficacy of twenty five molecules of plant origin for their anti-*Candida albicans* activity. Among the screened, cinnamaldehyde, piperidine, citral, furfuraldehyde and indole were potent inhibitors of growth and viability. Natural molecules like eugenol, anisaldehyde and methyl eugenol are reported to inhibit *C. albicans* growth, through ergosterol inhibition (Ahmad et al. 2010; Sheikh et al. 2011). The indirect effect of a flavonoid, epigallocatechin-3-gallate (EGCG) is known to alter ergosterol profile in *C. albicans* (Navarro-Martinez et al. 2006)*.* Two structurally related compounds, carvacrol and thymol are reported to exhibit fungicidal activity through disruption of ergosterol synthesis (Ahmad et al. 2010). Earlier reports have shown that β- asarone, an active principle *A. calamus* rhizome, alter sterol profile in *C. albicans* (Rajput & Karuppayil 2012). Among the tested, seven were found to alter ergosterol profile in *C. albicans,* where, fufuraldehyde and citral were the best even at sub-inhibitory concentrations*.* There was complete blockage of ergosterol synthesis at the MIC values. Studying the effect of these molecules at various sub-inhibitory concentrations on the sterols of *C. albicans* showed that they act in a dose dependent fashion to decrease the ergosterol content. Cinnamaldehyde, piperidine and indole were also found to be potential inhibitors of ergosterol biosynthesis. Although monoterpenes act by disrupting the microbial cytoplasmic membrane; however, specific mechanisms involved in the antimicrobial action of monoterpene remain poorly understood. β-pinene, an active component of pine oil, is known to affect the yeast membrane functions (Uribe et al. 1985). In our study, two structural isomers, α- and β- pinene (monoterpenes), showed similar pattern of ergosterol biosynthesis inhibition. This data indicate that structurally related compounds may have a common target to act. Our findings suggest that like fluconazole, molecules altering sterol profile may exert their antifungal activity through inhibition of ergosterol biosynthesis. The selective cytotoxic behavior of these molecules hints at their affinity for this particular specific target and hence, its biosynthetic pathway. Our study showed that most of the ergosterol inhibitors were fungicidal in its action. The *Candida*-cidal activity of molecules might be due to direct damage to the cell membrane. Exact mode of action of these effective molecules need to be elucidated.

## Conclusions

Naturally occurring plant molecules, fufuraldehyde, cinnamaldehyde and citral, could be lead molecules for fungal-specific drug development.

## Materials and methods

### Media, chemicals and culture conditions

*Candida albicans*, ATCC 90028 (MTCC 3017), strain was obtained from the Institute of Microbial Technology, Chandigarh, India, and was maintained on Yeast–Peptone–Dextrose (YPD) agar slants at 4°C. Sabourauds Dextrose Broth (SDB) and YPD media were purchased from Hi-Media Laboratories Ltd., Mumbai, India. All pure molecules were purchased from Sigma Aldrich chemicals, Pvt. Ltd., Bangalore, India and Hi-Media Laboratories Ltd., Mumbai, India.

### Growth assay

The susceptibility study was carried out by the standard methodology M27-A2 with slight modifications (Rajput & Karuppayil 2012). Briefly, various concentrations of molecule(s) or drug were prepared in SDB medium by double dilution in 96 well plates. Each well contained an inoculum of 1 × 10^3^ cells ml^-1^ and the final volume of SDB medium maintained in each well was 200 μl. The wells without addition of molecules served as control. Microplates were incubated at 35°C for 48 hours and read spectrophotometrically at 620 nm using a microplate reader (Multiskan EX, Thermo Electron Corp. USA). The lowest concentration of the molecule which caused fifty percent reduction in the absorbance compared to that of control was considered as Minimum Inhibitory Concentration (MIC).

### Minimum fungicidal concentrations

To determine the minimum *Candida*-cidal concentrations of the molecules, cells from wells containing the MICs and higher concentrations were selected. An aliquot of 10 μl cell suspension from each well was spread on YPD agar plates. The plates were incubated for 24 h at 30°C and observed for the presence of colonies. Minimum Fungicidal Concentration (MFC) was considered as the lowest concentration killing 99.9% cells (Zore et al. 2011)

### Ergosterol extraction and quantitation

A single *Candida* colony from an overnight Sabouraud dextrose agar plate culture was used to inoculate 50 ml of Sabouraud dextrose broth for control and for various concentrations of molecules. The cultures were incubated for 16 hours and harvested by centrifugation at 2,700 rpm (856 × g) for five min. The net weight of the cell pellet was determined. Three milliliters of 25% alcoholic potassium hydroxide solution was added to each pellet and vortex mixed for one min. Cell suspensions were transferred to sterile borosilicate glass screw-cap tubes and were incubated in an 85°C water bath for one hour. Following incubation, the tubes were allowed to cool. Sterols were then extracted by addition of a mixture of one ml of sterile distilled water and 3 ml of *n*-heptane followed by vigorous vortex mixing for 3 min. The heptane layer was transferred to a clean borosilicate glass screw-cap tube and stored at −20°C. Prior to analysis, 0.6 ml aliquot of sterol extract was diluted fivefold in 100% ethanol and scanned spectrophotometrically between 240 and 300 nm with a spectrophotometer (Shimadzu UV-Visible Spectrophotometer). The presence of ergosterol and the late sterol intermediate 24(28) dehydroergosterol (DHE) in the extracted sample resulted in a characteristic four-peaked curve. The absence of detectable ergosterol in extracts was indicated by a flat line. A dose-dependent decrease in the height of the absorbance peaks was evident and corresponded to decreased ergosterol concentration. Ergosterol content was calculated as a percentage of the wet weight of the cell by the following equations:

where *F* is the factor for dilution in ethanol and 290 and 518 are the E values (in percentages per centimetre) determined for crystalline ergosterol and 24 (28) DHE, respectively (Arthington-Skaggs et al. 1999).

### Statistical analysis

All the experiments were performed on three independent occasions. Values mentioned are the mean of triplicate observations and standard deviation from the mean was calculated. Percent decrease in ergosterol content at MICs of molecules as compared to control was analyzed using Student’s *t* test (Graphpad prism 5.0 software), and *p* <0.05 was considered statistically significant.
